# Sound Frequency Representation in the Auditory Cortex of the Common Marmoset Visualized Using Optical Intrinsic Signal Imaging

**DOI:** 10.1523/ENEURO.0078-18.2018

**Published:** 2018-05-07

**Authors:** Toshiki Tani, Hiroshi Abe, Taku Hayami, Taku Banno, Naohisa Miyakawa, Naohito Kitamura, Hiromi Mashiko, Noritaka Ichinohe, Wataru Suzuki

**Affiliations:** 1 Ichinohe Neural System Group, Laboratory for Molecular Analysis of Higher Brain Functions, RIKEN Brain Science Institute, Wako, Saitama 351-0198, Japan; 2 Department of Ultrastructural Research, National Institute of Neuroscience, National Center of Neurology and Psychiatry, Kodaira, Tokyo 187-8502, Japan; 3 Department of Biotechnology and Life Science, Graduate School of Engineering, Tokyo University of Agriculture and Technology, Koganei, Tokyo 184-8588, Japan; 4 Department of Otorhinolaryngology, University of Pennsylvania, Philadelphia, PA 19104; 5 Department of Functional Brain Imaging Research, National Institutes of Quantum and Radiological Science and Technology, Chiba, Chiba 263-8555, Japan

**Keywords:** ECoG, histology, marmoset, optical imaging, sound frequency, tonotopy

## Abstract

Natural sound is composed of various frequencies. Although the core region of the primate auditory cortex has functionally defined sound frequency preference maps, how the map is organized in the auditory areas of the belt and parabelt regions is not well known. In this study, we investigated the functional organizations of the core, belt, and parabelt regions encompassed by the lateral sulcus and the superior temporal sulcus in the common marmoset (*Callithrix jacchus*). Using optical intrinsic signal imaging, we obtained evoked responses to band-pass noise stimuli in a range of sound frequencies (0.5–16 kHz) in anesthetized adult animals and visualized the preferred sound frequency map on the cortical surface. We characterized the functionally defined organization using histologically defined brain areas in the same animals. We found tonotopic representation of a set of sound frequencies (low to high) within the primary (A1), rostral (R), and rostrotemporal (RT) areas of the core region. In the belt region, the tonotopic representation existed only in the mediolateral (ML) area. This representation was symmetric with that found in A1 along the border between areas A1 and ML. The functional structure was not very clear in the anterolateral (AL) area. Low frequencies were mainly preferred in the rostrotemplatal (RTL) area, while high frequencies were preferred in the caudolateral (CL) area. There was a portion of the parabelt region that strongly responded to higher sound frequencies (>5.8 kHz) along the border between the rostral parabelt (RPB) and caudal parabelt (CPB) regions.

## Significance Statement

In this study, we examined functional organizations for sound frequency representation in the core, belt, and parabelt regions in the marmoset using optical intrinsic signal imaging (OISI). In addition to the auditory areas in the core region, the mediolateral (ML) in the belt region continuously represented a range of frequencies from low to high, with tonotopic organization, which was symmetric to that in the primary (A1) area in the core region about the border between the A1 and ML. The posterior and anterior belt regions represented mainly high and low frequencies, respectively. The parabelt region contained a distinct region with preference for high frequencies. These results suggest that the functional organization unique to each auditory region would process sound information specific to this species.

## Introduction

Frequencies of sounds (voice, environmental sound, etc.) are represented in a systematic manner, e.g., tonotopic organization, in the auditory cortex. In areas with tonotopic organization, cells distribute over a cortical surface such that their preferred frequencies are continuously aligned from low to high. This organization has been found in the core region of the auditory cortex in humans and non-human primates (Formisano et al., 2003; Petkov et al., 2006; Humphries et al., 2010; Baumann et al., 2013). This area contains three smaller areas: the primary (A1), rostral (R), and rostrotemporal (RT) areas (Kaas and Hackett, 2000). The sound frequency preference gradient is reversed at the border between A1, R, and RT. This organization was revealed using sound frequency preference mapping, which is used to visualize the preferred frequency of cells on the cortical surface (Petkov et al., 2006; Bendor and Wang, 2008; Striem-Amit et al., 2011). Although previous studies have reported that the belt region also has a sound frequency preference gradient in macaque monkeys (Rauschecker and Tian, 2004; Petkov et al., 2006), the two-dimensional relationships between the individual areas in the belt regions are still unclear. The representation of sound frequency preference has not as yet been sufficiently studied in the parabelt regions. Because major parts of the auditory area are located in the lateral sulcus in humans and macaque monkeys, it is difficult to investigate the functional organization of the entire auditory area in detail. Furthermore, histologic areal demarcation in the auditory cortex is necessary to study the correspondence between functional organization and individual cortical areas. This has not been performed in most previous studies mapping the functional organization of this region.

The common marmoset (*Callithrix jacchus*) has well-developed vision and vocal communication and has been used in studies of the visual-related cortex (Mitchell et al., 2014; Hung et al., 2015; Suzuki et al., 2015a, 2015b; Miyakawa et al., 2017) and the auditory cortex (Eliades and Wang, 2008a, 2008b; Nelken et al., 2014; Agamaite et al., 2015; Miller et al., 2016; Abe et al., 2017). Because marmosets have lissencephalic and small brains, most areas of the auditory cortex in their brains are exposed on the cortical surface. This enables the relatively easy simultaneous imaging of these areas (Tokuno et al., 2009; Paxinos et al., 2012). Taking advantage of this fact, we investigated the cortical representation of sound frequency in a major portion of the auditory area of a small New World monkey, the common marmoset. Nishimura et al. (2017) have performed optical imaging using a voltage-sensitive dye in the common marmoset and obtained function signals in response to sound frequency. However, they did not perform histologic studies. It was thus difficult to conclusively demonstrate a rigorous relationship between the imaging data and the auditory area in that study. Here, we visualized the functional organization of the entire auditory cortex using optical intrinsic signal imaging (OISI), which is used to measure cortical responses with higher spatial resolution (20–30 µm) than that of other functional techniques [e.g., functional magnetic resonance imaging (fMRI): ∼1 mm; Cohen, 1973, Bonhoeffer and Grinvald, 1996]. We were thus able to directly compare the functional signals to the histologic areal demarcation data. We obtained evoked responses to band-pass noise stimuli in a range of sound frequencies (0.5–16 kHz) in anesthetized adult animals and revealed functional organizations by mapping the preferred frequency in each area in the core, belt, and parabelt regions of the auditory cortex.

## Materials and Methods

Experiments were performed using three adult common marmosets (one male and two females; body weight: 300–400 g). The surgical procedure, optical imaging, and electrocorticogram (ECoG) recording were approved by the Institutional Animal Research Committee at RIKEN (No. H13-B040) and the Experimental Animal Committee of the National Institute of Neurology and Psychiatry and were performed in accordance with the Guiding Principles for the Care and Use of Animals in the Field of Physiologic Science of the Japanese Physiologic Society.

### Surgery

The surgeries were conducted under anesthesia induced by an intramuscular injection of ketamine hydrochloride (Ketalar, 25 mg/kg) following an intramuscular injection of atropine sulfate (0.15 μg/kg). The animal was artificially ventilated with a mixture of 70–50% N_2_O, 30–50% O_2_, and 1.0–2.0% isoflurane. Electrocardiograms, end-tidal CO_2_, and rectal temperature were monitored continuously.

A head holder and a recording chamber (inside diameter: 18 mm) were implanted on the skull, and a craniotomy and a durotomy were performed inside the recording chamber to expose the auditory cortex for the OISI and the ECoG recordings.

### Acoustic stimuli

All of the acoustic stimuli were generated using MATLAB (MathWorks) and saved in uncompressed audio file format (100-kHz sampling frequency, 16 bits, mono). The sound delivery system was calibrated with 1/2-octave band-passed noises ranging from 0.5–16 kHz (1/2-octave step; 80-dB sound pressure level) using a noise meter (Sound level meter NL-27, RION). The sound stimuli were presented by a speaker system (BSSP10, iBUFFALO) placed 57 cm in front of the animal’s head.

### OISI

Following the surgeries, OISI was conducted under anesthesia maintained with an intravenous infusion of remifentanil (Ultiva, 0.1 μg/kg/min). During the recordings, muscular paralysis was induced with rocuronium bromide (Eslax, 13 μg/kg/min, i.v.). The recording chamber was filled with 0.5% agarose gel and sealed with a glass coverslip to minimize the movement of the brain due to respiration and cardiovascular pulses. A charge-coupled device (CCD; GRAS-03K2M-C, forward-looking infrared, Integrated Imaging Solutions Inc.) with 640 × 480-pixel resolution and 14-bit depth was used to obtain images reflected from the cortical surface. The cortex was illuminated by a band-pass filtered (535 ± 30 nm) halogen lamp through two fiber optic bundles. The CCD camera was focused on a plane 600 µm below the cortical surface (approximately layer 3) to avoid blood vessel artifacts, although the noise from the blood vessels at 535-nm wavelength was too high to completely remove the artifacts. The imaging data were acquired at a frame rate of 30 Hz during 10-s trials in which the 2-s auditory stimuli were presented from a loudspeaker starting 2 s after recording onset. The 2-s auditory stimulus was composed of the band-passed noises, with 100-ms on and 100-ms off. The stimulus duration of 100-ms is ordinary for electrophysiological experiments and was repeated 10 times in a trial to evoke strong responses.

The stimulus set consisted of ½-octave band-passed noises centered at 11 frequencies (0.5, 0.7, 1, 1.4, 2, 2.8, 4, 5.7, 8, 11.3, and 16 kHz). Each stimulus was presented 20–25 times in pseudorandom order with 20-s interstimulus intervals.

### ECoG recordings

Using the OISI results as a guide, we placed a multi-contact ECoG electrode (E32-1000-30-200, NeuroNexus) on the auditory cortex. The distances between neighboring contacts were 1 mm. The neural signals were amplified, band-pass filtered between 0.3 and 3 kHz, and stored at a 25-kHz sampling resolution into a TDT signal processing system (RZ2; Tucker-Davis Technologies). The digital time-stamps of the acoustic stimulus presentations were also stored.

### Data analysis

Intrinsic optical signals evoked by the stimuli were defined based on changes in the backscattered light following auditory stimulus presentation. The change at position *(x, y)* of an image at time t, $\frac{\Delta R}{R}$ *(x, y, t)*, was obtained using the following equation:

$$\frac{\Delta R}{R}\left(x,y,t\right)=\frac{{R\left(x,y,t\right)–〈R(x,y)〉}_{pre}}{{〈R(x,y)〉}_{pre}}$$

where *R(x, y, t)* is the backscattered light (pixel luminance value). The bracket indicates the mean of the backscattered light at position *(x, y)* for the 2-s period before the stimulus presentation. The changes were further averaged across the 20-25 presentation times for each stimulus. The wavelength of 535 nm used for illumination is an isosbestic point for absorption of oxy- and deoxyhemoglobin. Thus, following neural activation, the optical signal decreased due to the change in cerebral blood flow containing oxy- and deoxyhemoglobin.

To evaluate how the sound stimuli used in this study were effective for each auditory cortical area, the magnitude of the optical signals was calculated using the following equation:

$$Mag\left(x,y\right)=\underset{i=11}{\max}\left|\frac{{R_{i}\left(x,y\right)–〈R(x,y)〉}_{pre}}{{〈R(x,y)〉}_{pre}}\right|$$

where *R_i_(x, y)* was the backscattered light to i-th sound frequency averaged 4 s after stimulus onset.

The frequency preference map was created by calculating the expected values of the 11 sound frequencies for each pixel:

$$Pref\left(x,y\right)=\sum_{i=1}^{11}\frac{\Delta R}{R_{i}}\left(x,y\right)\times \frac{f(i)}{11}$$

where $\frac{\Delta R}{R_{i}}\left(x,y\right)$ is the optical signal to i-th sound frequency averaged 4 s after stimulus onset and *f(i)* is the i-th sound frequency (i = 1, …, 11). Note that the preferred sound frequency was a continuous real number.

The significant biased number of pixels that responded to a specific sound frequency in each area was evaluated using a bootstrap method. The preferred sound frequency was quantized to 10 steps (0.5–0.7, 0.7–1, 1–1.4, 1.4–2, 2–2.8, 2.8–4, 4–5.7, 5.7–8, 8–11.3, and 11.3–16 kHz). The number of pixels with the preferred sound frequency that were randomly placed in the auditory cortex was counted for each auditory area and iterated 1000 times. When the ratio of the original number to the pixels in each auditory area for a specific preferred sound frequency was >95% of the distribution of the ratio for the sound frequency with random sampling, the area was considered to have significantly biased representation for the specific sound frequency. We conducted the same analysis in each auditory region.

### Histology

After the end of the recording, an amount of sodium pentobarbital leading to an overdose (Somnopentyl; 75 mg/kg) was injected intraperitoneally, and the animals were intracardially perfused with saline followed by 4% paraformaldehyde (PFA). The brain was removed and post-fixed in 4% PFA overnight. The brain was then sequentially cryoprotected in 10%, 20%, and 30% sucrose in 0.1 M phosphate buffer.

The brains were coronally sectioned at a thickness of 50 µm using a freezing microtome (SM2010R, Leica). The processed sections were mounted on silan-coated slide glasses. For histologic processing, brain sections from one marmoset were divided into two series and mounted on glass slides. Odd-numbered sections underwent myelin staining, while even-numbered sections underwent Nissl staining. The staining was performed after we obtained fluorescent images from the sections. Brain sections from another marmoset were divided into three series. Two series of sections underwent myelin and Nissl staining, respectively. The third series of sections underwent fluorescent imaging after staining with DiI. Brain sections from third marmoset were divided into four series. Three series of sections underwent myelin, Nissl, and DiI staining. The fourth series of sections were left as reserve. All of the mounted sections were scanned using a digital slide scanner (NanoZoomer 2.0-HT, Hamamatsu Photonics K.K.; 20× objective, 455 nm/pixel). The tissues were silver-stained for myelin using an auto-metallographic technique described elsewhere (Pistorio et al., 2006). The sections stained for myelin were dehydrated in graded ethanol solutions, immersed in xylene, and cover-slipped in DPX (Sigma-Aldrich Co or Merck).

**Figure 1.**
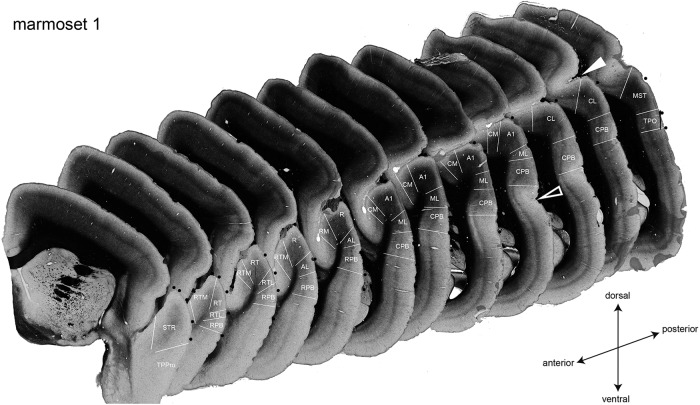
Coronal sections from the left hemisphere of a marmoset stained for myelin and the areal demarcation of the auditory cortical areas. Auditory cortical areas are defined by the myelin structure. The white lines indicate the areal borders. The black filled circles represent positions of blood vessels. The filled and open white arrowheads indicate the lateral sulcus and the superior temporal sulcus, respectively.

To delineate the areal borders in the auditory cortex based on the myeloarchitecture and cytoarchitecture in the coronal sections, we referred to previously published literature (Aitkin et al., 1986; de la Mothe et al., 2006b; Paxinos et al., 2012). For example, myelin was densest in the A1, and the borders between the A1 and neighboring areas could be easily defined in the coronal sections (Fig. 1). The large blood vessels on the cortical surface in the coronal sections were used as landmarks and the positions of the areal borders were identified with reference to the blood vessels. Subsequently, the blood vessels in the coronal sections were superimposed on the image obtained by the CCD camera used in the OISI experiment. The areal borders were reconstructed on the image and the sound frequency preference maps. Regions of interest were delimited by the histologically determined areal border.

## Results

### Responsiveness to band-pass noise stimuli in each auditory area in the core, belt, and parabelt regions

**Figure 2.**
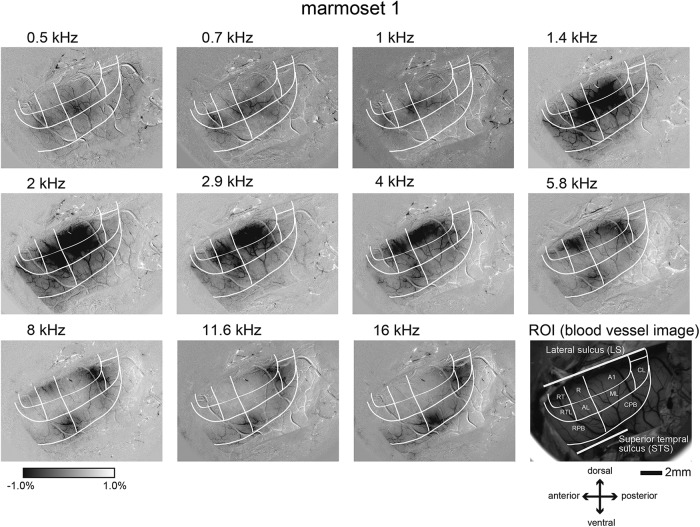
Optical responses to sound frequencies in the auditory cortex of the marmoset shown in Figure 1. A single condition map was reconstructed based on the optical signal changes in response to sound frequencies of 0.5, 0.7, 1, 1.4, 2, 2.9, 4, 5.8, 8, 11.6, and 16 kHz. The response magnitude is indicated by the gray scale shown at the left bottom. The black and white regions indicate the highest and lowest response magnitudes, respectively. The white lines represent the histologically defined areal borders of the auditory cortex.

Intrinsic optical signals evoked by band-pass noise stimuli were clearly observed in the core, belt, and parabelt regions in all three marmosets. The spatial locations of the optical signal distribution in response to the 11 sound frequencies with centers of 0.5, 0.7, 1, 1.4, 2, 2.9, 4, 5.8, 8, 11.6, and 16 kHz systematically changed on the cortical surface in different manners in the core, belt, and parabelt regions (Fig. 2). In the core region, when the sound frequency gradually changed from low to high, the corresponding responsive region continuously moved on the cortical surface. The regions responsive to stimuli with low to high frequencies mapped on the cortical surface from the antero-ventral to postero-dorsal direction in A1 and RT, and from the postero-ventral to antero-dorsal direction in R. A complete set of sound frequencies (from low to high) seemed to be represented in each area (A1, R, and RT). These results are consistent with those of previous electrophysiological studies performed in marmosets, in which unit recordings performed to map the best sound frequency showed a similar direction of continuous changes and complete representation of sound frequencies in the core region (Aitkin et al., 1986; Bendor and Wang, 2008).

In the mediolateral (ML) area of the belt region, the regions responsive to sound stimuli increasing in frequency from low to high continuously mapped in the antero-dorsal to postero-ventral direction. The remaining areas of the belt region, however, had biases for low or high frequencies. The regions responsive to lower frequencies (1.4–2.9 kHz) extended over the border between ML and A1. The regions responsive only to low frequencies were found in the anterolateral (AL) and rostrotemplatal (RTL) areas, and those responsive only to high frequencies were found in the caudolateral area (CL). In the parabelt region, there was no continuous mapping of the frequencies to the cortical surface. A region strongly responsive to high frequencies (>5.8 kHz) was present around the border between the rostral parabelt (RPB) and caudal parabelt (CPB) area. This is consistent with the findings of previous electrophysiological studies in macaque monkeys, in which multi-unit recordings suggested that cells in the parabelt region which preferred high frequency clustered in the superior temporal gyrus (Kajikawa et al., 2015).

**Figure 3.**
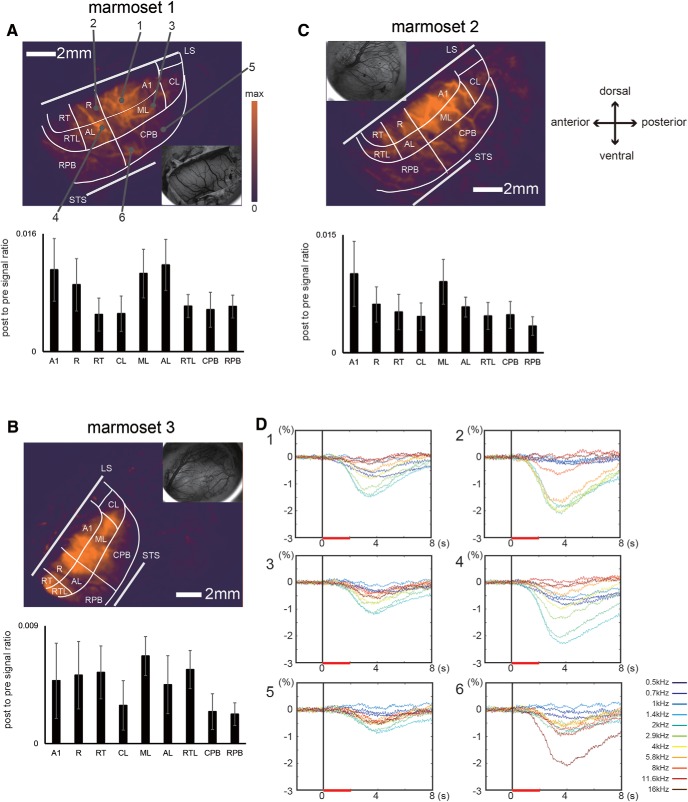
Response magnitude maps and the mean response magnitude in each auditory area for the three marmosets. ***A–C***, top, Response magnitude maps reconstructed based on maximum evoked optical signals for sound frequencies of 0.5, 0.7, 1, 1.4, 2, 2.9, 4, 5.8, 8, 11.6, and 16 kHz for each marmoset. The color indicates the maximum signal magnitude at each pixel according to scale bar shown in ***A***. Blood vessel images for each map are shown in the inset. Bottom, The mean response magnitude and SD in each auditory area. LS and STS represent the lateral sulcus and superior temporal sulcus, respectively. ***D***, Time course of optical signals in response to each stimulus frequency in the six regions indicated in ***A***. The colors of the lines correspond to the optical signal evoked for each frequency. The vertical black lines and horizontal red lines represent stimulus onset and the stimulus presentation period, respectively.

Although the band-pass noise stimuli used in this study evoked intrinsic optical signals in each region, their response properties were different among the auditory cortical areas. Figure 3*A–C* shows the response magnitude map (top) and the mean response magnitude in each area (bottom) for the three marmosets. Optical responses to the band-pass noise stimuli, which were estimated based on the magnitudes of the optical signals (Mag(x, y)) averaged over each area, were strong in the A1, R, ML, and AL regions, and weak in the RT, RTL, and CL regions in all marmosets (Table 1). In the parabelt region, the mean response magnitude was generally low in all marmosets (Table 1), although there were some regions with high response magnitudes, e.g., regions around the border between RPB and CPB. The time courses of the optical signals evoked in response to each sound frequency at the six positions on the response magnitude map are shown in Figure 3*D*. There was clear selectivity for sound frequency, even at positions with small amplitudes.

**Table 1.** The magnitude of the optical signals % (mean ± SD) in each auditory area

Subject	A1	R	RT	CL	ML	AL	RTL	CPB	RPB
Marmoset 1	1.1 ± 0.4	0.9 ± 0.4	0.5 ± 0.2	0.5 ± 0.2	1.1 ± 0.3	1.2 ± 0.3	0.6 ± 0.2	0.6 ± 0.2	0.6 ± 0.2
Marmoset 2	0.5 ± 0.3	0.5 ± 0.2	0.5 ± 0.2	0.3 ± 0.2	0.7 ± 0.1	0.4 ± 0.2	0.6 ± 0.1	0.2 ± 0.1	0.2 ± 0.1
Marmoset 3	1.0 ± 0.4	0.6 ± 0.2	0.5 ± 0.2	0.5 ± 0.2	0.9 ± 0.3	0.6 ± 0.1	0.5 ± 0.2	0.5 ± 0.2	0.3 ± 0.1

### Organization of sound frequency preference

**Figure 4.**
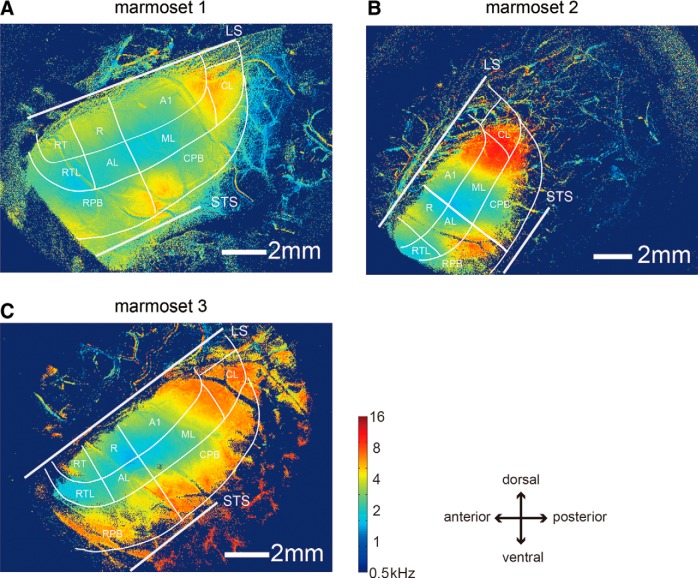
Sound frequency preference maps for the three marmosets. ***A–C***, Sound frequency preference maps reconstructed from averaged responses for all sound frequencies for the three marmosets. The color indicates the preferred frequency at each pixel according to the color code. LS and STS represent the lateral sulcus and superior temporal sulcus, respectively.

A frequency preference map in which the optical signals were averaged across the 11 sound frequencies at each pixel (*pref(x, y)*, defined in the materials and methods section) was used to estimate the representation of sound frequency in the auditory cortical areas (Fig. 4*A–C*). In the core region, a gradient of sound frequency preference was observed for all frequencies with the exception of those lower than 1.4 kHz. This gradient was reversed at A1/R and R/RT borders. No regions with preference for stimuli with frequencies higher than 8 kHz were observed at the R/RT border. This is likely because this region is within the lateral sulcus (Bendor and Wang, 2005, 2008). Therefore, with the exception mentioned above, the three complete sets of sound frequencies are thought to be represented in the A1, R, and RT regions.

In the belt region, there was only one area (ML) with a representation of the complete set of frequencies, with the exception of frequencies lower than 1.4 kHz. The topographical organization in this region was symmetric to that in A1 about the border between A1 and ML. In AL, the distribution of the preferred frequencies varied among three of the animals. There was a region with preference for high frequencies that protruded from the parabelt in two animals (Fig. 4*B*,*C*). The RTL mainly contained areas with preference for low frequencies and the CL had preference for high frequencies in all animals. There was a region that strongly responded to higher sound frequencies (>5.8 kHz) located around the middle part of the parabelt region in the rostro-caudal direction. This region was located mainly in the CPB in marmoset 1 and 3 and in the RPB in marmoset 2. In marmoset 1 and 3, regions with preference for high frequencies also existed in the anterior part of the RPB, which was distinct from the regions preferring high frequencies at the border between RPB and CPB. These anterior regions of the RPB were not found in marmoset 2, probably because the recording area did not include the anterior part of the RPB in this animal. These results indicate that the organization of sound frequency preference had different spatial arrangements among the core, belt, and parabelt regions, with some variance between individual animals.

**Figure 5.**
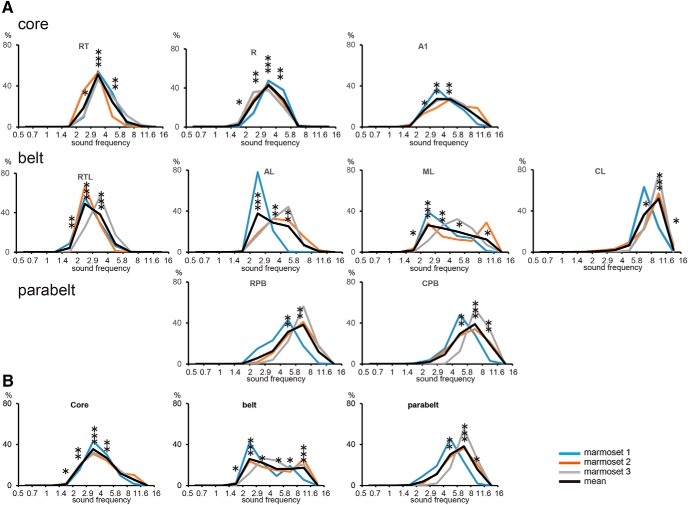
Distribution of preferred sound frequencies in each auditory cortical area. A: relative areas with preference for sound frequencies of 0.5–0.7, 0.7–1, 1–1.4, 1.4–2, 2–2.9, 2.9–4, 4–5.8, 5.8–8, 8–11.6, and 11.6–16 kHz. ***B***, Relative areas with preference for different sound frequencies in the core, belt, and parabelt regions. The pale blue, orange, and gray lines represent the three individual marmosets, and the black line represents the mean value for the three marmosets. The number of asterisks represent the number of animals with significantly biased representation in response to the sound frequency.

To quantify the sound frequency preference described above, we counted the number of the pixels for each preferred sound frequency in each auditory area and calculated the ratio of this number to that of all pixels in each area (Fig. 5*A*). The number of pixels used for each area in this analysis has been summarized in Table 2. If the ratio was widely and equally distributed along the preferred sound frequency range in an area, then the area was thought to represent a complete set of sound frequencies. As indicated in Figure 2, wide ranges of preferred sound frequencies, with the exception of frequencies lower than 1.4 kHz, were observed in A1, R, RT, and ML. However, there was a slight preference for high sound frequencies in R and RT, as shown in Figure 2. The distribution of the ratio was slightly biased toward low sound frequencies in RTL. The distribution was biased toward higher frequencies in CL, CPB, and RPB.

**Table 2.** The number of pixels for each defined auditory area

Subject	A1	R	RT	CL	ML	AL	RTL	CPB	RPB
Marmoset 1	14169	6961	4539	6690	11398	6180	6153	18074	18897
Marmoset 2	9417	5290	2018	5092	9072	5320	2247	16184	8828
Marmoset 3	12616	6660	3496	6709	11482	4935	5172	21342	22588

We compared the preferred sound frequencies among the core, belt, and parabelt regions (Fig. 5*B*). Complete sets of sound frequencies, with the exception of frequencies lower than 1.4 kHz, were observed in both the core and belt regions. In the core regions, the sound frequency preference tended to have a peak in the middle frequency range, probably due to less preference for high frequencies in R and RT. In the parabelt regions, the distribution of sound frequencies was biased toward high frequencies. These results suggest that the core and belt regions contain representations of a wide range of sound frequencies, while the parabelt region may have specialized areas to process mainly high sound frequencies.

### Comparison between optical and electrical signals

**Figure 6.**
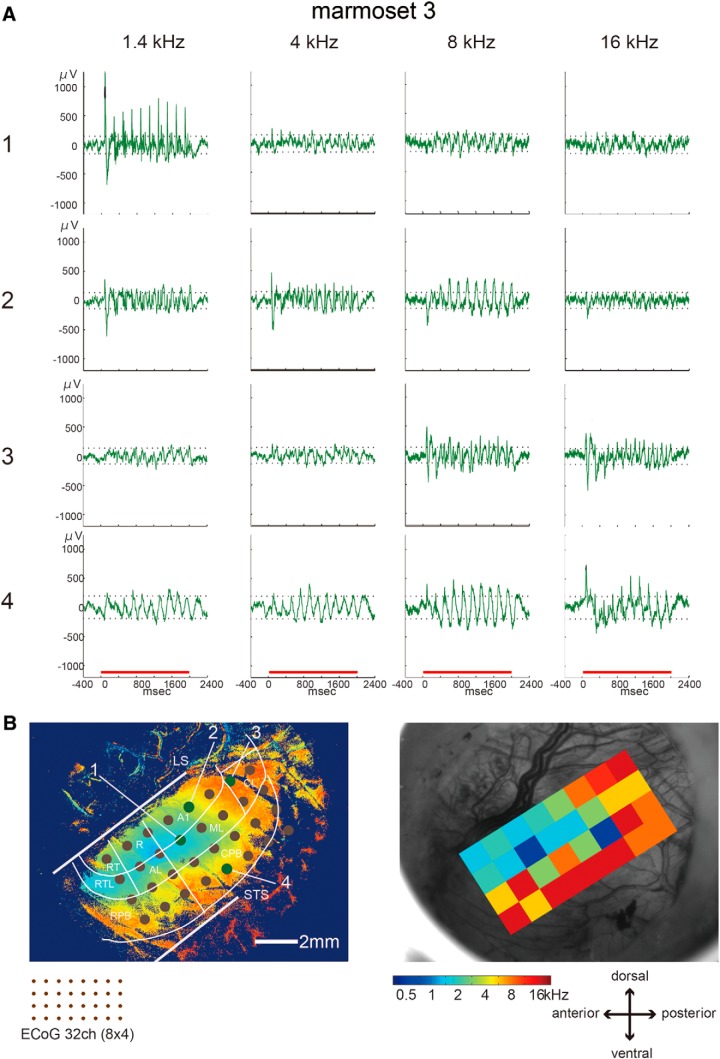
Electrical responses to band-pass noise stimuli. ***A***, ECoG responses to sound frequencies of 1.4, 4, 8, and 16 kHz at four contacts. The horizontal red lines represent the stimulus presentation period. ***B***, left, Electrode positions of the ECoG device on the sound frequency preference map made based on OISI in Figure 4*C*. Green circles represent the contact positions shown in ***A***. The brown circles correspond to the remaining contacts. LS and STS represent the lateral sulcus and superior temporal sulcus, respectively. Right, Sound frequency preference map reconstructed based on the maximum ECoG responses to all sound frequencies. Color indicates the preferred sound frequency at each contact according to the color code.

Finally, to confirm that the optical responses to sound frequencies reflected the neuronal responses, we compared the optical signals and the electrical response to the same sound stimuli in one animal (marmoset 3). Figure 6*A* shows an example of a local field potential in marmoset 3 at four ECoG contacts evoked by the band-pass noise stimuli used in the optical recording experiments. The local field potentials at positions 1 and 4 showed large responses to low and high frequencies, respectively. We constructed sound frequency preference maps with maximum responses to the 11 sound frequencies at all ECoG contacts. The sound frequency preference map for the ECoG recording was consistent with that for OISI (Fig. 6*B*).

## Discussion

**Figure 7.**
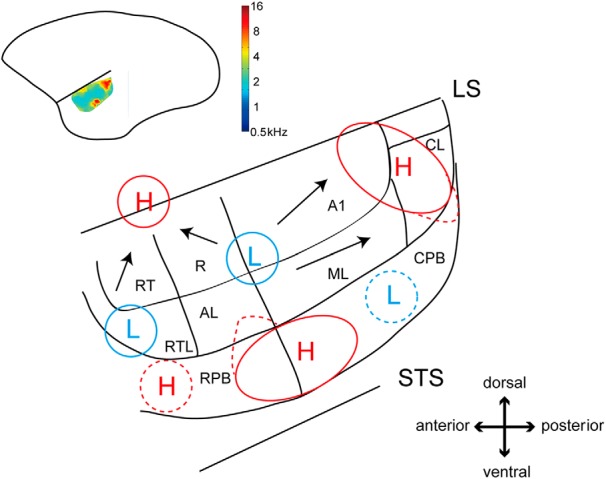
Sound frequency preference map for common marmosets. The circles surrounding H and L indicate regions with preference for high and low frequencies, respectively. The arrows indicate the direction of frequency preference change from low to high. The dotted region represents regions requiring further study. LS and STS represent the lateral sulcus and superior temporal sulcus, respectively. The color map in the simplified schematic of the marmoset cortex in the inset represents the sound frequency preference map.

In this study, we investigated the functional organization of sound frequency representation in a large area encompassed by the lateral sulcus and the superior temporal sulcus, including the core, belt, and parabelt regions in a small New World monkey, the common marmoset. We used OISI with reference to histologic areal demarcation. The auditory cortical areas A1, R, and RT in the core region and ML in the belt region responded to a range of sound frequencies from low to high with tonotopic organization. In the belt region, the RTL responded mainly to low frequencies, while the CL responded to high frequencies. Thus, the core and belt regions overall represented a complete set of the sound frequencies from low to high. In the parabelt region, the sound frequency preference was biased toward high sound frequencies. We have summarized our results in a sound frequency preference map for common marmosets (Fig. 7).

### Optical signals evoked by band-pass noise stimuli

We used band-pass noise stimuli with low to high sound frequency to evoke the cortical responses. Although the band-pass noise stimuli were simple sounds, optical signals to the stimuli were evoked not only in the core and belt regions, but also in the parabelt region. Electrophysiological studies have shown that neurons in the core region of the monkey auditory cortex respond to pure tones with various sound frequencies, as well as band-pass noise stimuli (Aitkin et al., 1986; Bendor and Wang, 2005, 2008; Kajikawa et al., 2005, 2011; Philibert et al., 2005). A fMRI study has also shown that these stimuli activate the core and belt regions of the auditory cortex in humans and monkeys (Petkov et al., 2006; Leaver and Rauschecker, 2016). The response magnitudes of intrinsic signals to band-pass noise stimuli were larger in the A1 and R areas of the core region and the ML and AL areas of the belt region than in the other auditory cortical areas. The A1 and R regions have focal topographic connections to the auditory thalamus with tonotopic organization. The A1 sends outputs to the ML and AL (de la Mothe et al., 2006a, 2006b, 2012a, 2012b). Thus, spectral analysis of acoustic stimuli might be massively processed in the A1, R, ML, and AL.

Although sound frequencies lower than 1.4 kHz evoked optical signals in line with the tonotopic organization shown in Figure 2, their contributions to the functional organization seemed to be small (Fig. 4). The ECoG results indicated that even sound stimuli lower than 1.4 kHz clearly elicited neuronal activity, as shown in Figure 6*B*. Thus, the insensitivity of the optical signals to low sound frequencies would be reflected in OISI. This is a limitation of the methods used here. We used the wavelength of 535 nm for illumination to the cortex which was reported the effectiveness for auditory optical imaging (Ojima et al., 2005). Noise, however, from the blood vessels with this wavelength was quite larger than that with the other wavelength, e.g., 630 nm. Further studies are necessary to map complete preferred frequency distribution including the low sound frequency.

In the parabelt region, although the response magnitude was low in general, there were small regions with higher response magnitudes around the RPB/CPB border. There was a tendency toward preference for high frequencies in these regions (Figs. 3, 4). Interestingly, relatively simple sound stimuli activated the parabelt region, which was thought to be a higher auditory area in macaque monkeys (Kaas and Hackett, 2000). This suggests that acoustic stimuli with high frequencies can be analyzed more precisely in this higher auditory area.

Using the simple band-passed noise stimuli, the sound frequency preference map was successfully reconstructed even for the auditory cortical areas with weak optical signals: the RT of the core region, the RTL and CL of the belt region, and the areas of the parabelt region. This was due to the high signal amplitude and signal-to-noise ratio, with the relatively high spatial resolution of OISI. Several studies using fMRI in macaque monkeys have mapped the preferred frequency in the core and belt regions, but not in the parabelt region with weak signals to the simple stimuli, which would be averaged in voxels with low spatial resolution. Voltage sensitive dye imaging with lower signal amplitude (1/10–1/100 of that of OISI) detected signals in the core and belt regions but not in the parabelt region of common marmoset, although it had better temporal resolution than OISI (Nishimura et al., 2017).

### Comparison of sound frequency representation among core, belt, and parabelt regions

The core region of the auditory cortex in primates contains tonotopic maps (Formisano et al., 2003; Petkov et al., 2006; Humphries et al., 2010; Baumann et al., 2013). This was shown in the marmoset using electrophysiological studies (Aitkin et al., 1986; Bendor and Wang, 2005, 2008). Our results obtained in the common marmoset were consistent with those reported for other primate species. Specifically, the preferred frequency gradually changed from low to high in the antero-ventral to postero-dorsal direction in the A1 and RT, and in the postero-ventral to antero-dorsal direction in R. Regions with preference for higher frequencies were not clearly observed in R or RT, probably because these regions extended into the lateral sulcus, as reported in previous studies (Bendor and Wang, 2005, 2008). The regions around the intersection of the A1, R ML, and AL areas had the lowest sound frequency preferences. It is thought that the gradual change in sound frequency preference from low to high starts at this intersection. Bendor and Wang (2005) suggested that the pitch center region in non-human primates where cells responsive to pitch exist is located around the regions with preference for the lowest frequencies.

It is as yet not well known whether tonotopic maps exist outside the core region in primates. Our results showed that there was tonotopic organization only in the ML area of the belt region. This organization was symmetric to that in A1 about the border between A1 and ML. The preferred frequency gradually changed from low to high in the antero-dorsal to postero-ventral direction. There was no tonotopic organization in the other areas of the belt region. The sound frequency preference was biased toward lower frequencies in the RTL and toward higher frequencies in the CL. We did not observe a region with preference for low frequencies, which is inconsistent with the previous findings of Petkov et al. (2006). When compared to the results of fMRI studies reporting the gradual change in frequency preference in the belt region in macaque monkeys (Petkov et al., 2006), our results further clarify the fine functional structure of the belt region with sub-areal scale. We were unable to investigate the medial part of the belt region in the lateral sulcus using OISI in this study. The use of fMRI with high spatial resolution, ECoG with high density electrodes, or new functional imaging techniques would be necessary to elucidate the sound frequency representation in the caudomedial, rostromedial, and rostrotempmedial areas in the medial belt region.

No tonotopic organization was found in the parabelt region, which contained the island-like region with preference for high frequencies (>5.8 kHz) around the border between the RPB and CPB. The spatial positions of the regions responsive to high frequencies varied among the different animals. We found regions with preference for high frequencies in the anterior part of the RPB. These regions had response magnitudes lower than that found in the RPB/CPB border. Regions with preference for high frequencies also exist at the border between RPB and CPB in macaque monkeys (Kajikawa et al., 2015). However, in contrast to the findings of this study, there is the gradient of sound frequency preference from the border to the anterior RPB and to the posterior CPB (Kajikawa et al., 2015). The difference in functional organization between the two species might be attributable to their vocal frequency ranges and their living environments.

The sound frequency preference map in the core region is a common functional structure among the primate species (Aitkin et al., 1986; Formisano et al., 2003; Rauschecker and Tian, 2004; Bendor and Wang, 2005; Petkov et al., 2006; Humphries et al., 2010; Baumann et al., 2013; Nishimura et al., 2017). This presence of this auditory cortical structure in primates might be reflected by their abundant vocal communication compared to other mammals. On the other hand, the spectral features of vocal sounds among different primate species (e.g., the main frequency contained in vocal sounds of marmosets is ∼8 kHz) might cause the structural differences in sound frequency representation in the belt and parabelt regions, as discussed above.

## Synthesis

Reviewing Editor: Tatyana Sharpee, The Salk Institute for Biological Studies Decisions are customarily a result of the Reviewing Editor and the peer reviewers coming together and discussing their recommendations until a consensus is reached. When revisions are invited, a fact-based synthesis statement explaining their decision and outlining what is needed to prepare a revision will be listed below. The following reviewer(s) agreed to reveal their identity: Shuang Li, Andrew Dykstra

This study reports measurements of the tonotopic organization of auditory cortical fields in the common marmoset, an increasingly important animal model for auditory processing. The authors find, for the first time, the existence of tonotopic maps outside the core region in primates.

It would be helpful to discuss the relative advantages and limitations of the intrinsic signal imaging compared to other techniques. For example, intrinsic signal imaging provides higher resolution than fMRI and can survey broader regions of cortex than measurements made using voltage sensitive dyes. It would also be helpful to discuss why auditory processing should be similar across all of primates regardless of the folding patterns for the cortex, making the results obtained in the marmoset relevant to other primate species.

Reviewer 1:

1. Line 65: It will be helpful to justify OISI as high spatial resolution. For example, give the spatial resolution of the current approach and compare with previous approaches.

2. Line 107: Which cortical layer (depth) is imaged and why? From figure 3 A, C, blood vessel structure is readily visible from the imaged results. This is an indication of imaging from the superficial layer, which could further interfere with spatial resolution and conclusion.

3. Line 108 - 111: Protocol design should be based on the property of simulation sound and imaging signal dynamics. Justification will be necessary.

4. Line 140: The original sample size to perform bootstrapping from, i.e., the number of pixels for each defined area, is important to evaluate the method.

5. Line 146: The author should give detailed explanation how each histological measurement being used in the final results

6. Line 186: The results session is in general not specific enough. For example, in line 186, how are the results consistent with cited literature exactly?

7. Line 191: Please be consistent with the notation low frequency, lower frequencies

Reviewer 2:

-the 2nd sentence of the intro starts with ‘therefore’, causally linking spectral features of sound and how those features are represented. This is an unwarranted and teleological argument.

-no units in Figure 2. Also, it's not immediately apparent (at least for anyone who is unfamiliar with the details of OISI) why a lesser optical signal should be a higher response magnitude, as is stated in the legend for the figure. Please clarify, at least briefly.

-in the legend for Fig 7: “The dotted region and the arrow represent regions requiring further study.” I don't see the arrow that the authors refer to in the figure.

-the authors should consider using different color maps for responsiveness (e.g. fig 3) and frequency specificity (figs 4, 6, and 7).

-Lines 212-214: “A frequency preference map in which the optical signals were averaged across the 11 sound frequencies at each pixel was used to estimate the representation of sound frequency in the auditory cortical areas.”

I don't understand this sentence. How is a frequency preference map derived from signals averaged across frequencies?

-In all figures in which data from the three individuals are shown, it would help the reader if the respective panels were labeled to reflect this. In figure 5, a legend with color labels (I.e. marmoset 1, 2, and 3) would be helpful.

-Lines 254-255: “Finally, to confirm that the optical responses to sound frequencies reflected the neuronal responses, we compared the optical signals and the electrical response to the same sound stimuli in an animal.”

‘an’ → ‘one’, and maybe include a parenthetical stating which animal (with reference to figures 3 and 4, i.e. the same animal as figure 4c).

-Lines 293-294: “The ECoG results indicated that even sound stimuli lower than 1.4 kHz clearly elicited neuronal activity, as shown in Figure 6A.”

Figure 6A shows no such thing, as the lowest frequency shown there is 1.4 kHz. Did the authors mean to refer to the ECoG map in the right-sided panel of Figure 6B instead?
